# Traits correlate with invasive success more than plasticity: A comparison of three *Centaurea* congeners

**DOI:** 10.1002/ece3.4080

**Published:** 2018-06-30

**Authors:** Daniel Montesinos, Ragan M. Callaway

**Affiliations:** ^1^ Division of Biological Sciences and the Institute on Ecosystems The University of Montana Missoula Montana; ^2^ Centro de Investigaciones sobre Desertificación – CIDE (CSIC, UV, GV) Carretera Moncada‐Náquera Moncada Spain; ^3^ Centre for Functional Ecology Department of Life Sciences University of Coimbra Calçada Martim de Freitas Coimbra Portugal

**Keywords:** biogeography, competition, nutrient availability, phenotypic plasticity, relative distance plasticity indexes, trait shifts

## Abstract

The importance of phenotypic plasticity for successful invasion by exotic plant species has been well studied, but with contradictory and inconclusive results. However, many previous studies focused on comparisons of native and invasive species that co‐occur in a single invaded region, and thus on species with potentially very different evolutionary histories. We took a different approach by comparing three closely related *Centaurea* species: the highly invasive *C. solstitialis,* and the noninvasive but exotic *C. calcitrapa* and *C. sulphurea*. These species have overlapping distributions both in their native range of Spain and in their non‐native range of California. We collected seeds from 3 to 10 populations from each region and species and grew them in common garden greenhouse conditions to obtain an F1 generation in order to reduce maternal effects. Then, F1 seeds were grown subjected to simulated herbivory, variation in nutrient availability, and competition, to explore plasticity in the responses to these conditions. We found little variation in phenotypic plasticity among species and regions, but *C. solstitialis* plants from California produced more biomass in competition than their Spanish conspecifics. This species also had the highest relative growth rates when in competition and when grown under low nutrient availability. Noninvasive congeners produced intermediate or opposite patterns.

## 1 INTRODUCTION

Phenotypic plasticity is the ability of a genotype to express different phenotypes in response to different environmental conditions (West‐Eberhard, 1989). Phenotypic plasticity is thought to broaden the ecological niches of species and thus play an important role in biological invasions (Daehler, 2003; Richards, Bossdorf, Muth, Gurevitch, & Pigliucci, 2006; Zenni, Lamy, Lamarque, & Porté, 2014). This may be particularly important in disturbed environments where environmental variation is frequent (Daehler, 2003). Adaptive plasticity in colonizing populations may also provide a temporal buffer prior to directional and local selection (Ghalambor, Mc Kay, Carroll, & Reznick, 2007; Pigliucci, 2005). However, empirical evidence for the role of plasticity in invasive success is contradictory, with some meta‐analyses and reviews finding no difference in plasticity between invasive and noninvasive species (Godoy, Valladares, & Castro‐Díez, 2011; Godoy, Valladares, & Castro‐Díez, 2012; Palacio‐López & Gianoli, 2011), with others finding substantial differences (Daehler, 2003; Davidson, Jennions, & Nicotra, 2011).

Phenotypic plasticity is often considered an alternative to adaptation, but the possibility that increased or decreased plasticity could also be selected for is usually overlooked (Matesanz, Gianoli, & Valladares, 2010). Furthermore, high plasticity levels are not necessarily associated with fitness gains (Davidson et al., 2011) and reaching a particular balance between plasticity and local trait adaptation could be key to invasive success (Liao, D'Antonio, Chen, Huang, & Peng, 2016). Rapid local adaptation by colonizing or invading species is an alternative to phenotypic plasticity for coping with new environmental conditions. A recent review of 31 reciprocal transplant experiments considering 362 traits showed that 52% of the studied traits were not plastic, and that among the 48% of traits showing some kind of plasticity, 31% of it were nonadaptive. These results suggest that local adaptation might be a more common response to variable environments than adaptive phenotypic plasticity (Palacio‐López, Beckage, Scheiner, & Molofsky, 2015). A second meta‐analysis (Liao et al., 2016) suggested that phenotypic plasticity explained a higher proportion of phenotypic variation in clonal, self‐compatible, and perennial species, whereas local adaptation explained more phenotypic variation for annual species. Liao et al. (2016) also assessed the relative importance of plasticity and adaptation trait by trait, and suggested that phenotypic plasticity played a more important role in traits related to fecundity and biomass allocation, whereas local adaptation was more important in traits related to phenology. Regardless, it is clear that both local adaptation and phenotypic plasticity can act synergistically in the successful colonization of new habitats (Liao et al., 2016).

Studies of plants collected from populations in their native and non‐native regions, and grown in a uniform environment, often report that individuals from the non‐native range grow faster, achieve larger final size, compete more intensely, and produce weaker defenses against specialist herbivores (Bossdorf et al., 2005; Colautti, Maron, & Barrett, 2009; Hawkes, 2007; van Kleunen, Weber, & Fischer, 2010; Ridenour, Vivanco, Feng, Horiuchi, & Callaway, 2008). Phenotypic plasticity could minimize the effect of selective differences among genotypes, thus hiding important genetic variation from selection in non‐native ranges of exotic plants (Baquedano, Valladares, & Castillo, 2008). As selective adaptation is often correlated with invasive success, higher plasticity levels could be detrimental in some conditions for long‐term invasive success. In a comparison of multiple sets of invasive and noninvasive congeneric species, invasive species grew larger and performed better than noninvasives, but they were similar in plasticity over a range of phenotypic traits (Godoy et al., 2012). In contrast, *Centaurea melitensis* plants from the non‐native range were higher in phenotypic plasticity for several traits than plants from the native range (Moroney, Rundel, & Sork, 2013). Previous comparisons of *C. solstitialis* with other *Centaurea* species found no clear relationship between invasiveness and phenotypic plasticity, but a higher tolerance to low nutrient availability for the invasive *C. solstitialis* (Muth & Pigliucci, 2007).

Previous studies have evaluated the role of phenotypic plasticity in exotic invasions by comparing native and exotic species within the non‐native ranges of the exotics. Also, most experimental assessments have used individuals grown from seed without an effort to reduce maternal effects. We have learned much from these approaches, but focusing on related invasive and noninvasive congeners, collected from both native and non‐native ranges, and for which maternal effects are reduced, might contribute even more to our understanding of exotic invasion. Here, we selected three closely related *Centaurea* congeners (*C. solstitialis, C. calcitrapa, C. sulphurea*) with overlapping distributions in their native and non‐native ranges, but with very different degrees of abundance and local dominance (or success) in each of those regions. The overlapping distributions in both the native and non‐native ranges of Spain and California make it highly likely that our populations had experienced similar environmental and selective pressures in the past. We also grew seeds from different individuals and populations in common greenhouse conditions to obtain an F1 generation of individuals with reduced maternal effects. Three general factors affect the expression of phenotypic plasticity in plants: variation in the abiotic environment, variation in consumer pressure, and variation in the competing plant community (Callaway, Pennings, & Richards, 2003). Consequently, we designed experiments to assess the relative importance of phenotypic plasticity and trait variation in response to low and high nutrient availability, clipping, and competition in the context of differential invasive success.

## 2 METHODS

*Centaurea solstitialis, C. calcitrapa,* and *C. sulphurea* are three closely related species from the Jacea group in the *Centaurea* genus (Garcia‐Jacas et al., 2006). These species are related enough to hybridize when artificially crossed (Montesinos, Santiago, & Callaway, 2012). The three species have overlapping distributions and appear to have similar environmental requirements in both their native (Eurasia) and non‐native (North America) and thus share aspects of their evolutionary histories. *Centaurea solstitialis* is an annual herb native from Southern Europe and introduced into the Americas and Australia (Eriksen et al., 2014; Gerlach & Rice, 2003; Maddox, Mayfield, & Poritz, 1985). It was introduced into California (USA) at the latest in 1824 (Maddox et al., 1985), where it continuously increased its large‐scale distribution and population numbers, and where it has become an highly problematic invader (Eagle, Eiswerth, Johnson, Schoenig, & Cornelis van Kooten, 2007; Hay, Facelli, & Panetta, 2006). Genetic data indicate that *Centaurea solstitialis* has been introduced multiple times to California (Eriksen et al., 2014). *Centaurea calcitrapa* was probably introduced into California in approximately 1896 (Pitcairn, Young, Clements, & Balciunas, 2002; Robbins, 1940) and has naturalized since, but it is much less abundant and not invasive like *C. solstitialis*, sustaining only a moderate number of more or less stable populations. *Centaurea sulphurea* was introduced into California by 1923 at the latest (Muth & Pigliucci, 2006), has a highly restricted native range in Spain and Morocco, and exists in only a handful of naturalized populations in California (Gerlach & Rice, 2003). However, in Spain the Californian invader *C. solstitialis* is less common than the noninvasive *C. calcitrapa* (Garcia‐Jacas et al., 2006). *Centaurea sulphurea* is uncommon in both ranges (http://www.gbif.org).

In the summer of 2009, seeds of all three species were collected from fifteen different individuals from 3 to 10 different populations at each Spain and California (see list of the 45 populations in the Appendix S1). In 2010, seeds from each individual were grown in common conditions in a greenhouse with pollinators excluded, and with temperatures between 15 and 30°C and light supplemented by metal halide bulbs. Seeds were germinated and plants grown in pots with local soil from Missoula, Montana, until flowering, and then, manual cross‐pollinations were performed between individuals of the same population in order to obtain an F1 of seeds, potentially with reduced maternal effects (see detailed methodology at Montesinos et al., 2012). In 2010, we set up a common garden experiment with seeds from the F1 generation and germinated and grew full‐sibling seeds from the same parental individuals under four different environmental conditions. These were the control groups, clipping (removal of 50% of the leaves one month after germination) and no‐clipping, low and high nutrient availability, and one‐to‐one competition with *Bromus hordeaceus* (seeds purchased from S&S Seeds, Carpinteria, CA, USA). *Centaurea* and *Bromus* seeds were shown simultaneously in each competition pot. *Bromus hordeaceus* is an annual grass native to Europe that co‐occurs with the three *Centaurea* species in both their native and non‐native ranges.

Seeds were planted in 0.5‐L Ray Leach Inc. *Cone‐Trainers* pots in a 50:50 mix of 20–30 grit sand and soil from grasslands in the Missoula Valley (Montana) and watered every 1–2 days. Each experimental group had five individuals from five different maternal families from each of the 45 populations from all regions and species, replicated by each of the four experimental groups (control, no fertilization, simulated herbivory, and competition), totaling 900 replicates. Full‐sibling individuals belonging to the same maternal family from each population and region were compared for differences in response to each of the treatments. We fertilized all treatments except the nutrient‐limited treatment. Fertilization consisted of 100 ml of 1.16 g/L Scotts Miracle‐Gro (15:30:15 + micronutrients) every other week. Plants were grown for four months, harvested and dried at 70°C for 48 hr, and weighed.

We used total biomass to calculate simplified relative distance plasticity indexes (RDPI_S_), which are specifically designed to allow for statistical testing among different populations and species (Valladares, Sanchez‐Gomez, & Zavala, 2006). RDPI ranges from 0 (absence of plasticity) to 1 (maximum plasticity). We assessed plasticity, relative growth rate (RGR, calculated as increases in the maximum diameter of each rosette), and total biomass independently for each of the three different experimental groups using linear mixed‐effect models. These models were applied with the approach of Laird and Ware (1982) in R 3.1.2 (R Development Core Team, 2010) using the procedure *lme* of the package *nlme*, with plasticity (RDPI) as the study variable, species and region as fixed factors, and population nested within each region as random factor. Tukey post hoc tests were used to test for differences among each pair of species using the *glht* procedure in R's *multcomp* library.

## 3 RESULTS

**Figure 1:**
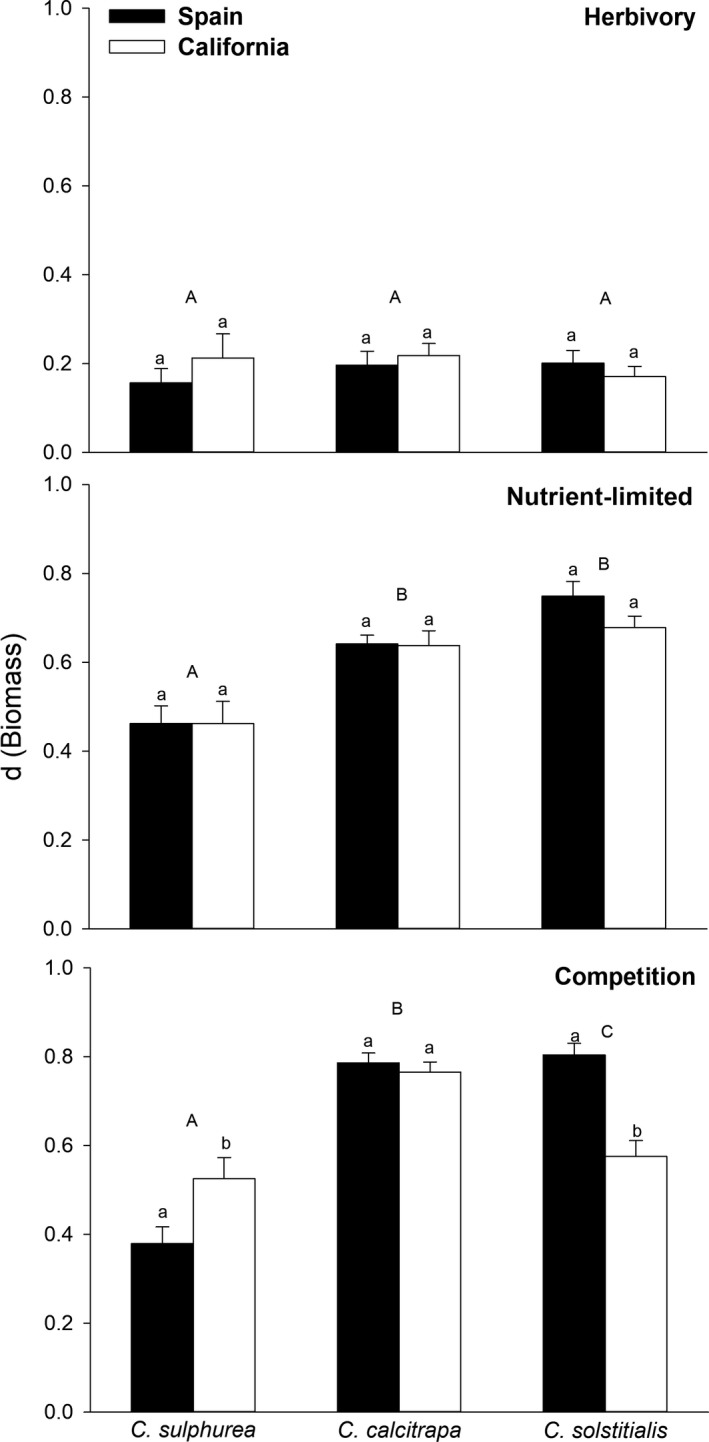
Inter‐regional differences for biomass phenotypic plasticity for each species (RDPI, mean ± *SE*), under three different treatments. Different uppercase or lowercase letters indicate statistically significant differences (*p* ≤ .05) among species or between regions, respectively

Individuals from the three species and the two regions did not differ in their plasticity in response to clipping, and plasticity in response to clipping was low in general (*F*_2,39_ = 0.78; *p* = .465; Figure 1). Variation in nutrient availability caused no differences in plasticity between the native and non‐native regions for any of the species (*F*_1,41_ = 0.69; *p *= .411). However, *Centaurea* species differed in their plastic responses to nutrients (*F*_2,42_ = 14.76; *p* ≤ .001). Post hoc tests showed that *C. sulphurea* was less plastic than the other two species (*z* = 4.11, *p* ≤ .001; and *z* = 5.48, *p* ≤ .001). Competition with *B. hordeaceus* triggered the highest plasticity values for all three *Centaurea* species. In addition, competition drove the largest differences between plants from native and non‐native regions (*F*_1,41_ = 4.18; *p* = .047) and among species (*F*_2,42_ = 16.33; *p* ≤ .001, and post hoc for each pair: *z* = 5.73; *p* ≤ .001; *z* = 3.75; *p* ≤ .001; *z* = −2.62, *p* = .023). This resulted in a significant interaction between regions and species (*F*_2,39_ = 8.94; *p* ≤ .001). In sum, there was higher plasticity in response to competition for Spanish *C. solstitialis* than Californian *C. solstitialis*, the opposite pattern for the *C. sulphurea,* and no differences between regions for *C. calcitrapa* (Figure 1).

**Figure 2:**
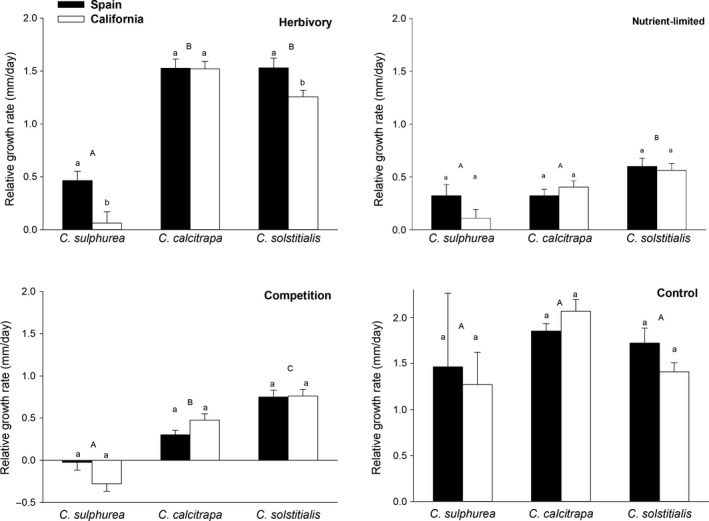
Relative growth rates of different *Centaurea* species subjected to four different treatments. Bars show mean ± *SE* values. Different uppercase or lowercase letters indicate statistically significant differences (*p* ≤ .05) among species or between regions, respectively

Relative growth rates (RGR) were similar for all species (*F*_2,35_ = 2.779; *p* = .076) and regions (*F*_1,35_ = 0.246; *p* = .321) in the control group; however, each of the three treatments resulted in different patterns between regions and among *Centaurea* species. Herbivory significantly reduced the growth rates of *C. sulphurea* and *C. solstitialis* from California when compared with their counterparts from Spain, but had no effect on the RGR of *C. calcitrapa* (*F*_species, 2,35_ = 82.229; *p* < .001; *F*_range, 1,39_ = 7.085; *p* = .011; *F*_species*range, 2,39_ = 2.633; *p* = .0846). *Centaurea sulphurea* had a lower RGR than the other two species (both post hoc tests *p* < .001). Low nutrient availability reduced RGRs for all species, but there was no significant difference between regions (*F*_1,38_ = 0.030; *p* = .862), and only nonfertilized *C. solstitialis* attained significantly faster RGRs than the other two species (*F*_2,38_ = 10.713; *p* < .001, both post hoc tests *p* < .002). Finally, competition did not result in significant differences in RGRs between regions (*F*_1,41_ = 0.229; *p* = .635), but all three species differed significantly from each other (*F*_2,41_ = 29.958; *p* < .001, all post hoc tests *p* < .001), with *C. sulphurea* under competition producing negative RGRs, *C. calcitrapa* intermediate RGRs, and *C*. *solstitialis* the highest RGRs (Figure 2).

**Figure 3:**
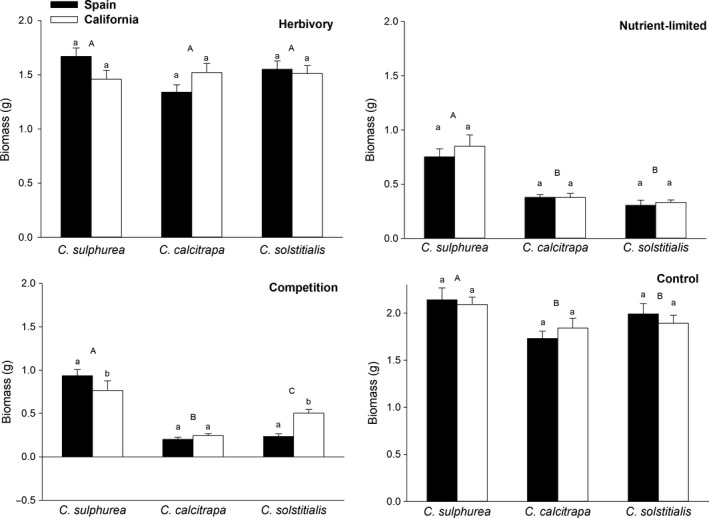
Total final biomass of different *Centaurea* species subjected to four different treatments. Bars show mean ± *SE* values. Different uppercase or lowercase letters indicate statistically significant differences (*p* ≤ .05) among species or between regions, respectively

Total biomass of control plants was marginally higher for *C. sulphurea* than for the other two species (*F*_2,39_ = 2.701; *p* = .079, all post hoc tests *p* ≤ .058), and there were no significant differences among regions for any species (*F*_1,39_ = 0.002; *p* = .964). High nutrient availability doubled the biomass of all species and increased the difference between *C. sulphurea* and the other two species, but otherwise treatment and region effects were similar to those of the controls (*F*_2,39_ = 43.172; *p* < .001, both post hoc tests *p* < .001), and regions (*F*_1,39_ = 0.467; *p* = .499). Clipping slightly reduced biomass compared to controls, and eliminated the differences in biomass between *C. sulphurea* and the other two species (*F*_2,39_ = 0.829; *p* = .444). There were no differences between regions (*F*_1,39_ = 0.087; *p* = .770). Again, competition drove the largest differences, with each species responding differently (*F*_2,39_ = 83.875; *p* < .001). *Centaurea calcitrapa* produced the lowest final total biomass in response to competition, *C. solstitialis* was intermediate, and *C. sulphurea* the highest (all post hoc tests *p* < .001). Interestingly, control *C. sulphurea* plants from California produced less biomass than their Spanish conspecifics, whereas in competition *C. solstitialis* from California accumulated more biomass than their Spanish counterparts (region *F*_1,39_ = 7.193; *p* < .011; interaction region × treatment *F*_2,39_ = 10.631; *p* < .001; Figure 3).

## 4 DISCUSSION

We found no strong relationships between phenotypic plasticity and invasive success for these three closely related *Centaurea* species. However, our results point to at least two potential characteristics that could promote the differential success of the invasive *C. solstitialis,* the ability to sustain high growth rates under nutrient‐limited conditions, and the ability of individuals from California to sustain high growth rates when experiencing competition.

In contrast, the least successful and abundant of the three species in the non‐native range of California, *C. sulphurea*, showed the opposite pattern for some of the same traits. These included the lowest growth rates when in competition and in response to simulated herbivory. The noninvasive *C. calcitrapa*, which occurs at intermediate abundance in California, showed no significant differences between Spain and California for any of the studied traits. Mean trait and plasticity values tended to be intermediate, or more similar to those of the invasive *C. solstitialis*. The exception was total biomass when in competition, where *C. calcitrapa* produced the lowest biomass of the three species.

Previous multispecies comparisons assessing biomass, survival, physiology, and leaf functional traits of co‐occurring native and invasive species suggest that phenotypic plasticity is not significantly higher for invasive plant species than for natives overall and that, regardless of plasticity, fitness‐related traits are more important than plasticity for invasive success in most cases (Godoy et al., 2011, 2012; Palacio‐López & Gianoli, 2011). In another similar meta‐analysis (Davidson et al., 2011), the invasive species considered were more phenotypically plastic overall, but such plasticity was not associated with fitness gains. This is consistent with the perspective that local adaptation is a more common mechanism than phenotypic plasticity allowing plants to deal with environmental variability (Palacio‐López et al., 2015). Several studies have reported different sets of local adaptations to different non‐native ranges for several traits of the same *Centaurea* species studied here (García, Callaway, Diaconu, & Montesinos, 2013; Graebner, Callaway, & Montesinos, 2012; Montesinos et al., 2012). Together with our results, this suggests that local adaptation may be more important than plasticity for dealing with the new environments of the non‐native ranges for these three *Centaurea* species.

Plasticity can be beneficial, but it can also have fitness costs that are directly related to the magnitude of plasticity shown by some species (Ghalambor et al., 2007; Relyea, 2002; Stinchcombe, Dorn, & Schmitt, 2004; Valladares, Gialoni, & Gómez, 2007). The potential costs of plasticity include maintaining the sensory mechanisms responsible for plastic responses, producing a phenotype different from that of a nonplastic individual, the genetic cost derived from the interaction between plasticity genes and other genes, developmental instability, and the costs derived from imprecise detection of environmental changes (DeWitt, Sih, & Wilson, 1998; Relyea, 2002). As a consequence, the costs of plasticity might significantly affect the evolution of optimal phenotypes (Relyea, 2002). Although experimental evidence is scarce and contradictory (DeWitt et al., 1998; Scheiner & Callahan, 1999), in some cases the lack of plasticity in dispersal‐related traits appears to enhance colonization by some invasive species (Brock, Weinig, & Galen, 2005).

The potential benefits of plasticity can also change depending on the stage of invasion. For example, a reciprocal transplant experiment in France and Canada assessing the physiology and leaf functional traits of two invasive *Acer* tree species found that invasive populations of one species presented strong trait generic differentiation and also greater plasticity than native populations. In contrast, the other *Acer* species was highly plastic but there were no traces of genetic differentiation between native and non‐native populations. This suggests that higher levels of phenotypic plasticity might be more important during the early stages of colonization than later in the invasion process (Lamarque, Lortie, Porté, & Delzon, 2014). In that regard, our results are similar, as the highly invasive *C. solstitialis* showed the lowest level of plasticity among the non‐native populations.

Other than plasticity, trait shifts by exotic species between the native and non‐native ranges can correlate with invasive success (Hierro, Maron, & Callaway, 2005; Maron, Vilà, Bommarco, Elmendorf, & Beardsley, 2004). Each of the three species we studied showed a number of trait shifts consistent with rapid local adaptation (García et al., 2013; Graebner et al., 2012; Hierro et al., 2009; Montesinos et al., 2012; Sotes et al., 2015; Xiao, Callaway, Graebner, Hierro, & Montesinos, 2016). However, these studies used plants originated from seed collected from the field and were thus exposed to confounding maternal effects related to the environmental conditions in which the populations were sampled. Here, the F1 generation of seeds we used should have had reduced maternal effects, as the parental generation was grown in common greenhouse conditions. Under these controlled conditions, damage from clipping resulted in strong reductions in RGRs in *C. sulphurea*, particularly for individuals from California, and in reductions in *C. solstitialis* individuals from California. However, differences in growth rates did not result in differences between regions or among species in total final biomass in the controlled conditions of the greenhouse. In previous studies (García et al., 2013; Graebner et al., 2012), *C. sulphurea* produced the most biomass but had the lowest relative growth rates when compared to the other two species. In this study, this did not occur for control individuals. Maternal effects in earlier studies may be responsible for this difference.

Exotic species frequently benefit, relative to natives, from high nutrient availability (Leishman & Thomson, 2005; Pyšek & Richardson, 2007). Consistently, we found that nutrient limitation reduced total biomass for all species, but the noninvasive *C. sulphurea* was not as affected by low nutrients as the other two species, perhaps due to its larger seeds (Graebner et al., 2012). Nevertheless, the invasive *C. solstitialis* sustained significantly higher growth rates than their congenerics even in low nutrient conditions, which is in agreement with a recent meta‐analysis comparing invasive and co‐occurring native species, where invasive species demonstrated significantly higher RGR than natives (van Kleunen et al., 2010).

The invasive *C. solstitialis* from California produced more biomass than their Spanish conspecifics. Overall, *C. solstitialis* individuals had the highest growth rates of the three species. In contrast, the noninvasive *C. sulphurea* plants from California were the most plastic and experienced the greatest reductions in biomass when in competition, providing a potential explanation for the minimal success of this naturalized species in its non‐native range.

Comparisons among invasive and noninvasive congeners with overlapping distributions in both their native and non‐native ranges suggested factors that might contribute to generally different patterns of success of the species in the non‐native range of California. Specifically, the highly invasive *C. solstitialis* was the most plastic in response to variation in nutrient availability, but with individuals from the non‐native range lower in plasticity than those from the native range. This is consistent with recent results reported by Dlugosch et al. (2015) suggesting that invasiveness of *C. solstitialis* in California could be explained by exploitation of water resources from deep soil layers which are usually unavailable to native species. We did not explore water limitation in our study, but water and nutrient limitation are connected in Mediterranean‐type ecosystems (Di Castri, 1981; Rodà, Mayor, Sabaté, & Diego, 1999).

Our study sheds light on the importance of plasticity and selection in colonization processes and illustrates the importance of comparing several traits a across a range of closely related species sharing both their native and non‐native ranges. This is in contrast to comparisons of species only in the invaded regions. The invasive and noninvasive species studied here shared some trait shifts, but the invasive species was able to excel in nutrient‐limiting environments and under competition. Our results suggested that plasticity might play a neutral or even negative role for the species we studied and at the stage of invasion we explored.

## CONFLICT OF INTERESTS

The authors declare that there are no conflict of interests.

## AUTHOR CONTRIBUTIONS

DM and RMC conceived the ideas and the experimental design, DM executed the experimental work and analyzed the data, and DM and RMC wrote the manuscript.
