# Supplementary material for: Traits correlate with invasive success more than plasticity: A comparison of three Centaurea congeners
Source: Ecol Evol. 2018 Jun 30;8(15):7378–85. doi: 10.1002/ece3.4080 (PMC6106188; doi:10.1002/ece3.4080)
Supplement: Supplementary file 1 [file ECE3-8-7378-s001.docx]

**Supporting information**

**Appendix S1**. Location of *Centaurea* populations for each of the three studied species on each of the two studied regions. Latitude and longitude coordinates are datum WGS84.
